# ENROLLING PARTICIPANTS WITH DEMENTIA IN A RURAL SETTING: GEISINGER RECRUITMENT STRATEGIES FOR D-CARE

**DOI:** 10.1093/geroni/igac059.1210

**Published:** 2022-12-20

**Authors:** Maya Lichtenstein, Pamela Borek, Ilene Ladd

**Affiliations:** Geisinger Health System, Wilkes Barre, Pennsylvania, United States; Office of Clinical Research Operations, Geisinger Wyoming Valley, Wilkes Barre, Pennsylvania, United States; Genomic Medicine Institute, Geisinger Health System, Danville, Pennsylvania, United States

## Abstract

Geisinger Health System serves a large, geographically stable population in central and northeast Pennsylvania and was chosen to participate in D-CARE due to its rural population, which is often under-represented. To reach potential participants, we used a multi-pronged recruitment approach including local radio, television, and newspaper advertisement; publication in a health system newsletter targeted to 55+; outreach to senior-care managers and primary care providers to encourage referrals; and leveraged an extensive electronic health record (EHR) database to identify a pool of almost 9,000 potential participants. Initial barriers to enrollment stemmed from geographic challenges and the dyad’s inability and/or unwillingness to travel long distances to lengthy in-clinic appointments. A post-pandemic transition to virtual visits helped ease the travel burden and increase study enrollment. Despite varied recruitment strategies, by far the most effective recruitment method remained direct referrals of patients and caregivers with whom the provider had discussed the D-CARE study.

